# Connectomic markers of disease expression, genetic risk and resilience in bipolar disorder

**DOI:** 10.1038/tp.2015.193

**Published:** 2016-01-05

**Authors:** D Dima, R E Roberts, S Frangou

**Affiliations:** 1 Centre for Neuroimaging Sciences and Social, Genetic and Developmental Psychiatry Centre, Institute of Psychiatry, Psychology and Neurosciences, King's College London, London, UK; 2 Department of Psychiatry, Icahn School of Medicine at Mount Sinai, New York, NY, USA; 3 Division of Brain Sciences, Imperial College London, London, UK

## Abstract

Bipolar disorder (BD) is characterized by emotional dysregulation and cognitive deficits associated with abnormal connectivity between subcortical—primarily emotional processing regions—and prefrontal regulatory areas. Given the significant contribution of genetic factors to BD, studies in unaffected first-degree relatives can identify neural mechanisms of genetic risk but also resilience, thus paving the way for preventive interventions. Dynamic causal modeling (DCM) and random-effects Bayesian model selection were used to define and assess connectomic phenotypes linked to facial affect processing and working memory in a demographically matched sample of first-degree relatives carefully selected for resilience (*n*=25), euthymic patients with BD (*n*=41) and unrelated healthy controls (*n*=46). During facial affect processing, patients and relatives showed similarly increased frontolimbic connectivity; resilient relatives, however, evidenced additional adaptive hyperconnectivity within the ventral visual stream. During working memory processing, patients displayed widespread hypoconnectivity within the corresponding network. In contrast, working memory network connectivity in resilient relatives was comparable to that of controls. Our results indicate that frontolimbic dysfunction during affect processing could represent a marker of genetic risk to BD, and diffuse hypoconnectivity within the working memory network a marker of disease expression. The association of hyperconnectivity within the affect-processing network with resilience to BD suggests adaptive plasticity that allows for compensatory changes and encourages further investigation of this phenotype in genetic and early intervention studies.

## Introduction

Bipolar disorder (BD) is characterized by mood dysregulation resulting in recurrent episodes of depression and mania with variable interepisode remission. BD remains one of the leading causes of disability worldwide across all age groups^1^ because of our incomplete understanding of its biological basis. This motivates efforts to characterize reliable biological markers of risk and resilience to BD. Identification of neurobiological mechanisms of resilience is of particular importance as it may offer clues for preventive interventions.

There is a strong genetic contribution to the etiology of BD, with estimated heritability between 60 and 85%.^2^ The genetic architecture of BD is complex and probably polygenic.^3^ As patients with BD and their unaffected relatives are likely to share some susceptibility genes, shared neuroimaging abnormalities are considered genetically driven markers of risk. Neuroimaging abnormalities present in patients but not in their relatives are considered markers of disease expression, whereas neuroimaging measures that differentiate unaffected relatives both from patients and unrelated healthy individuals are likely to represent markers of resilience.^4^

Structural and functional magnetic resonance imaging (MRI) studies have been extensively used to identify neural markers of disease expression, risk and resilience to BD. A trend toward larger whole-brain volumes has been observed in structural MRI studies comparing unaffected relatives with unrelated healthy controls,^5, 6^ in contrast to BD patients who show subtle but measurable reductions in whole-brain and regional gray matter volumes.^7, 8^ Functional MRI (fMRI) studies provide a richer source of information as they assess the regional mean signal changes (activation) and inter-regional interactions (connectivity) across distinct situational demands.^9, 10^ In BD, task-dependent activation and connectivity have been examined mostly in terms of affect processing and executive control, based on behavioral data that implicate dysfunction in these domains in patients and their relatives.^7, 11, 12^ Affect processing is known to involve multiple regions, notably the amygdala (AMG), ventral striatum and putamen and the ventral prefrontal (VPFC), ventral anterior cingulate (ACC) and insular cortices.^13^ Executive control comprises diverse functions supported by a common network that includes striatal structures, the dorsolateral prefrontal (DLPFC), dorsal ACC and parietal (PAR) cortices.^14^ In patients with BD, exaggerated activation during affective and executive tasks has been consistently observed in the AMG, insula and ventral ACC, whereas in unaffected relatives it is mostly confined to the insula.^5, 15, 16^ Patients show evidence of reduced PFC engagement during affective and non-affective tasks,^15, 17, 18, 19, 20^ while unaffected relatives show a trend toward PFC hyperactivation.^5, 21^ Changes in regional activation may reflect either inherent abnormalities or reactive responses to deficits elsewhere in the brain. Therefore, a network-level approach is required in order to further characterize neural markers of disease expression, risk or resilience to BD. At any given point, the connectomic features of a network are defined by the nature and degree of neural network disruption, the situational demands and the available network reserve.^22^ Increased situational demands within a network are typically met with increased connectivity; however, abnormally increased (hyper-) or decreased (hypo-) connectivity represents reactive responses to network disruption and, respectively, depend on the availability or loss of network reserve.^22^

In patients with BD, connectivity within the affect-processing networks is abnormally increased between subcortical regions^23, 24^ and in forward connections from subcortical to ventral PFC regions.^19, 25, 26, 27^ At the same time, regulatory input from the PFC to subcortical and posterior cortical regions appears reduced.^19, 28, 29, 30, 31^ Within the executive control network, patients with BD show diffuse hypoconnectivity affecting subcortical, mostly hippocampal and striatal, structures and key dorsal cortical regions in the DLPFC, ACC and PAR.^32, 33, 34, 35, 36, 37^ Thus, disease expression in BD appears to be associated with (a) hyperactivation and hyperconnectivity between affect-processing subcortical regions and (b) reduced regulatory input from the ventral PFC and the dorsal executive control network regions.

Studies in unaffected first-degree relatives have also found increased connectivity of subcortical and cortical affect-processing regions.^38^ However, relatives also appear to have compensatory hyperconnectivity between the DLPFC and either the VLPFC^35^ or the PAR cortex.^39^ These findings suggest that avoidance of overt disease expression (that is, resilience) may be associated with preserved network capacity that allows for compensatory connectivity changes. We tested this hypothesis by combining conventional statistical parametric mapping with dynamic causal modeling (DCM)^40^ of fMRI data to characterize activation and connectivity patterns in resilient relatives of patients with BD, patients wit BD and unrelated healthy participants, during facial affect recognition and working memory, two prototypical tasks of affect processing and executive control.

## Materials and methods

### Participants

A demographically matched sample of euthymic patients with BD (*n*=41), of their unaffected siblings (*n*=25) and healthy individuals (*n*=46), selected from the VIBES sample,^6, 19, 20, 34, 35, 41^ participated in the present study (Table 1). The sample included 17 patient-resilient sibling pairs, all from separate families. The diagnostic status of all participants was assessed using the Structured Clinical Interview for DSM-IV for Axis I diagnoses.^42, 43^ Patients fulfilled criteria for BD type I according to the Diagnostic and Statistical Manual of Mental Disorders, 4th edition, revised (DSM-IV).^44^ The first-degree relatives were carefully selected based on a strict definition of resilience detailed below. Unrelated healthy controls were selected based on the absence of family history and personal lifetime history of psychiatric disorders. In all participants, psychopathology was rated using the Hamilton Depression Rating Scale (HDRS),^45^ Young Mania Rating Scale (YMRS)^46^ and Brief Psychiatric Rating Scale (BPRS);^47^ current IQ was assessed using the Wechsler Adult Intelligence Scale 3rd Edition^48^ and general functioning with the Global Assessment of Function^44^ (GAF). To ensure that the patients were in remission, their psychopathology was assessed weekly over a period of 1 month prior to testing and at each assessment they scored below 7 in the HDRS and YMRS. Patients were also required to have remained on the same type and dose of medication for a minimum of 6 months. Although the level of symptomatology was very low, group differences were observed in HDRS (*P*=0.0001), YMRS (*P*=0.004) and BPRS total scores (*P*=0.0001); patients were more symptomatic than the other two groups (*P*<0.02). The BPRS, HDRS and YMRS scores were highly correlated (all *r*>0.73, *P*<0.0001). To avoid collinearity we used the total BPRS score as a covariate in subsequent analyses because, unlike the two other scales, it is applicable to nonclinical populations.

We employed strict criteria for resilience to minimize the likelihood of including relatives who may appear resilient because they have yet to manifest psychopathology or who had no evidence of expressed genetic traits. The peak period of risk for the onset of BD is between 16 and 30 years,^49^ whereas conversion rates thereafter are very low.^50^ Therefore, in this analysis we included relatives that (a) had passed though the peak risk period and (b) had no lifetime history of any psychopathology, assessed retrospectively at the time of scanning and prospectively at 4 years post-scanning, (c) expressed predisposition to BD in terms of abnormal ventral PFC–insula connectivity similar to that seen in patients.^35^

### Facial affect-recognition paradigm

Three negative facial emotions (fear, anger and sadness) were examined in a randomized order in three event-related experiments during a single acquisition session. In each experiment, 10 different facial identities (www.paulekman.com) depicting 150% intensity of a negative or neutral facial expression were presented in a pseudorandom order interspersed with a fixation cross. Each stimulus (affective and neutral faces; fixation cross) was displayed for 2 s and repeated 20 times. Participants were instructed to indicate whether the face had an emotional or a neutral expression. Response time and accuracy data were collected.

### Working memory paradigm

The N-back verbal working memory task was presented as an alternating block paradigm incorporating active conditions (1-, 2- and 3-back) and a baseline (0-back) condition. Participants were instructed to respond to target letters by button press. In the baseline condition, participants responded to the X letter. In the 1-, 2- and 3-back conditions participants responded when the letter currently presented matched the one presented in the preceding 1, 2 or 3 trials. There were 18 epochs in all, each lasting 30 s. Each letter was presented for 2 s. Performance was evaluated in terms of response time to target letters and accuracy.

### Image acquisition

Both anatomical and functional imaging data were acquired during the same session using a 1.5TGE Sigma. For the *facial affect-recognition* paradigm, 450 T2*-weighted MR images reporting blood-oxygen-level-dependent (BOLD) contrast were acquired (repetition time=2000 ms, echo time=40 ms, flip angle=70°, slice thickness=7mm, matrix size=64*64, voxel dimensions=3.75x3.75x7.7 mm). For the *working memory* paradigm, a total of 180 T2*-weighted MR volumes depicting BOLD contrast were acquired (repetition time=3000 ms, echo time=40 ms, flip angle=90°, slice thickness=3 mm, matrix size=64*64, voxel dimensions=3.75x3.75x3.30 mm).

A high-resolution T1-weighted structural image was acquired for each participant in the same session in the axial plane for co-registration (inversion recovery prepared, spoiled gradient-echo sequence; repetition time=18 ms, echo time=5.1 ms, flip angle=20°, slice thickness=1.5 mm, matrix size=256*192, field of view=240x180 mm, voxel dimensions=0.9375x0.9375x1.5 mm).

### Functional image processing

Conventional and DCM analyses were implemented using SPM8 (www.fil.ion.ucl.ac.uk/spm/software/spm8/). For both paradigms, fMRI images were realigned, normalized and smoothed using an 8-mm full-width-half-maximum Gaussian kernel.

For the *facial affect-recognition* paradigm, each participant's fMRI data from the three event-related experiments (fear, anger or sadness) were concatenated and vectors of onset representing correct responses were convolved with a canonical hemodynamic response function. Six movement parameters were also entered as nuisance covariates. The means of the three sessions as well as the transition at the end of each session were also modeled. For each participant, contrast images of affective>neutral faces were produced.

For the *working memory* paradigm, the smoothed single-participant's images were analyzed using the linear convolution model, with vectors of onset representing the experimental conditions (1-, 2- and 3 - back) and the baseline condition (0-back). Six movement parameters were also entered as nuisance covariates. Contrast images of the 3-back>baseline condition were produced for each participant.

### Conventional fMRI analysis

For each paradigm, contrast images were entered in a second-level random-effects analysis. The effect of group (patients, relatives and controls) was tested using a one-way analysis of variance with the BPRS total score as covariate. Suprathreshold clusters were identified using family-wise error correction of *P*<0.05, *k*>20. Stereotactic coordinates were converted from the MNI to the Talairach and Tournoux spatial array.^51^

### DCM analysis

**Figure 1:**
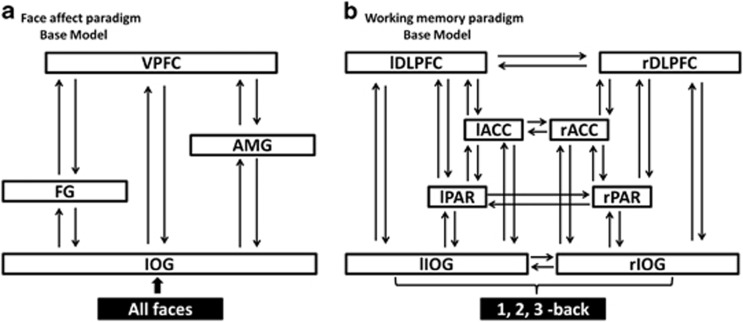
Dynamic causal models (DCM) architecture for bipolar disorder (BD) patients, their resilient relatives and healthy individuals. (**a**) Base model for the face affect paradigm. The model comprises four brain areas specified with bidirectional endogenous connections between all regions (inferior occipital gyrus=IOG, fusiform gyrus=FG, amygdala=AMG, ventral prefrontal cortex=VPFC; all located in the right hemisphere) and with a driving input of ‘all faces' into the IOG. (**b**) Base model for the working memory paradigm. An eight-area DCM was specified with bidirectional endogenous connections between all brain regions (lIOG, left IOG and rIOG, right IOG; lPAR, left PAR and rPAR, right PAR; lACC, left anterior cingulate cortex and rACC, right ACC; lDLPFC, left dorsolateral prefrontal cortex and rDLPFC, right DLPFC) in each hemisphere and lateral connections between homologous areas. Driving input of ‘1-, 2- and 3 -back' modeled into the left and right IOG.

DCM tests a set of models and, through Bayesian model selection, provides evidence in favor of one model relative to others. For each task we defined the relevant model space (that is, the set of models that are plausible) based on current best evidence regarding the neural circuitry that supports facial affect recognition and working memory. For the *facial affect-recognition paradigm,* previous studies implicate the inferior occipital gyrus (IOG), fusiform gyrus (FG), AMG and VPFC, most consistently on right.^52, 53, 54^ We therefore produced a basic 4-node DCM in the right hemisphere with endogenous connections between volumes of interest specified in the IOG, FG, AMG and VPFC. The main effect of 'all faces' was modeled as driving input to the IOG (Figure 1a). We then created all possible models derived through permutation of condition-specific responses (affective faces) on the forward coupling strength toward the VPFC. For the *working memory paradigm,* previous studies emphasize the bilateral involvement of the IOG, PAR, ACC and DLPFC.^55, 56^ We produced a basic 8-node DCM with endogenous connections between volumes of interest specified bilaterally in the IOG, PAR, ACC and DLPFC. The main effect of 'working memory' was modeled as driving input to the IOG (Figure 1b). We then created all possible models derived through permutation of condition-specific responses (3-back) on the coupling strength between nodes.

Seven models were produced for the *facial affect-recognition* paradigm and 32 for the *working memory* paradigm (Supplementary Information; Supplementary Table S1; Supplementary Figures S1 and S2). For each paradigm separately, models were compared using random-effects Bayesian Model Selection in SPM8 to compute exceedance and posterior probabilities for each group.^57^ To summarize the strength of effective connectivity and quantify its modulation, we used random-effects Bayesian Model Averaging to obtain average connectivity estimates across all models for each participant.^58^ Bayesian Model Averaging connections and modulations were extracted and tested in SPSS20 using analyses of variance or Kruskal–Wallis tests if data were not normally distributed.

## Results

### Behavioral data

Details on task performance are shown in Table 1. For *facial affect recognition*, there was a main effect of group on response time (*P*=0.004), with patients being slower than the other two groups (*P*<0.007), but not on accuracy (*P*=0.20). Conversely, in the *working memory* paradigm, there was a main effect of group on accuracy during the 3-back condition (*P*=0.004), with relatives outperforming the other two groups (*P*<0.003), but not on response time (*P*=0.10). Patients' medication type and dose did not affect their performance on either task (all *P*>0.40).

### Conventional fMRI analysis

#### Facial affect recognition

A group effect in the contrast affective>neutral faces was noted in the right ventral ACC and right superior frontal gyrus, where patients showed, respectively, increased and decreased activation compared with their relatives and unrelated controls (Supplementary Information; Supplementary Table S2).

#### Working memory

Group differences were noted only in the 3-back>0-back condition. BD patients showed reduced activation in the bilateral middle and inferior frontal gyrus, and increased activation in the right temporal gyrus and bilateral ACC compared with the other two groups. Resilient relatives showed higher activations in these areas compared with unrelated healthy controls (Supplementary Information; Supplementary Table S2).

Patients' medication type and dose did not affect any of the above results.

### DCM analysis

#### Facial affect recognition

**Figure 2:**
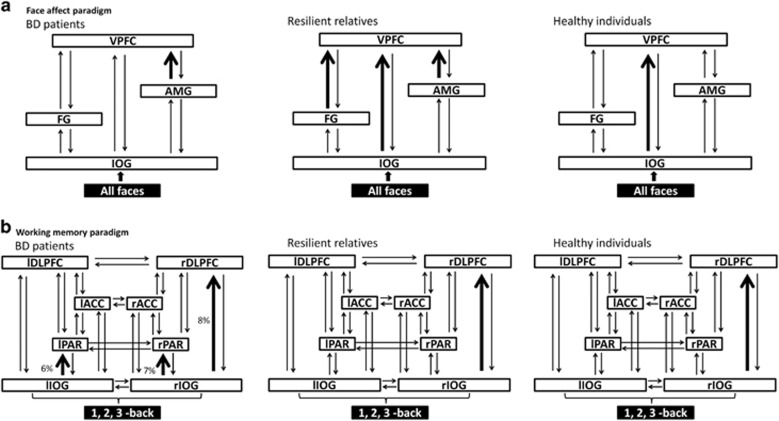
Results of dynamic casual modeling (DCM) model selection for bipolar disorder (BD) patients, resilient relatives and healthy individuals. (**a**) Optimal DCM model selection for the face affect paradigm. The models comprised a four-area DCM specified with bidirectional endogenous connections between the inferior occipital gyrus (IOG), fusiform gyrus (FG), amygdala (AMG) and ventral prefrontal cortex (VPFC), with a driving input of all faces into the IOG. Bold black arrows represent where facial affect modulation (corresponding to fearful, angry and sad faces) was placed in the winning model for each group. (**b**) Optimal DCM model selection for the working memory paradigm. The models compromised an eight-area DCM specified with bidirectional endogenous connections among the left IOG (lIOG) and right IOG (rIOG), the left parietal cortex (lPAR) and right PAR (rPAR), the left anterior cingulate cortex (lACC) and right ACC (rACC), the left dorsolateral prefrontal cortex (lDLPFC) and right DLPFC (rDLPFC), with a driving input of 1-, 2-, 3-back into the lIOG and rIOG. Bold black arrows represent where 3-back modulation was placed in the winning model for each group.

**Figure 3:**
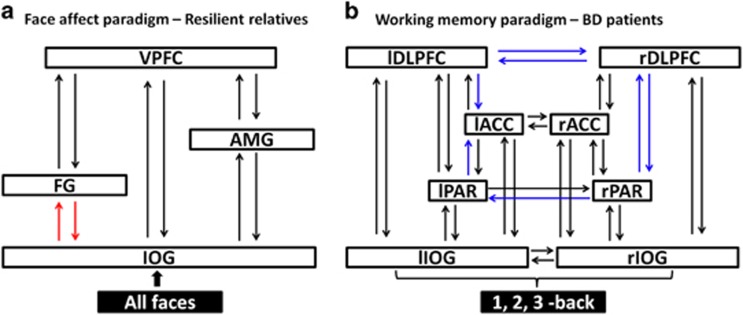
Group differences in effective connectivity within facial processing and working memory networks. (**a**) Alterations in effective connectivity within the facial processing network established by Bayesian model averaging across all models. The red arrows indicate significantly increased connectivity in resilient relatives of patients compared with patients and healthy individuals. (**b**) Alterations in effective connectivity within the working memory-processing network established by Bayesian model averaging across all models. The blue arrows indicate significantly reduced connectivity in BD patients compared with resilient relatives and healthy individuals.

The exceedance probabilities of all models are shown in Supplementary Table S3. The optimal models are shown in Figure 2a. In unrelated healthy individuals, the optimal model, with an exceedance probability of 41%, was the model that allowed facial affect to increase the strength of the forward connection from the IOG to the VPFC. In patients with BD, the optimal model, with an exceedance probability of 32%, allowed facial affect to increase the strength of the forward connection from the AMG to the VPFC. In resilient relatives, the optimal model, with an exceedance probability of 33%, allowed facial affect to increase the strength of the forward connections to the VPFC from the IOG, the FG and the AMG. Across all models, affect processing in patients compared with unrelated controls was associated with reduced connectivity between IOG and VPFC (*P*=0.02) but increased between AMG and VPFC (*P*=0.03). Across all models, there was a significant effect of group on the reciprocal endogenous connectivity between the IOG and the FG (*P*<0.04), which was higher in resilient relatives compared with both other groups (Figure 3a).

#### Working memory

The exceedance probabilities of all models are shown in Supplementary Table S3. The optimal models are shown in Figure 2b. Unrelated healthy individuals and relatives had the same optimal model with respective exceedance probabilities of 57 and 20%. This model allowed for the working memory load to increase the strength of the connection from the right IOG to the right DLPFC. No optimal model was identified in BD patients. The best model, but with an exceedance probability of 8%, was the same as that identified for relatives and controls. The second and third best models, with exceedance probabilities of 7% and 6%, respectively, allowed for the working memory load to increase the strength of the connection from the IOG to the PAR, either on the right or the left hemisphere. In these three models, duration of illness was negatively correlated with the memory load modulation of the forward connections from IOG to the DLPFC (*r*=−0.48; *P*=0.004) or to the PAR (left: *r*=−0.37; right: *r*=−0.42; *P*<0.01). Conversely, higher GAF scores were associated with greater working memory modulation of the forward connections from the right IOG to the right PAR (*r*=0.45, *P*=0.01) and to the right DLPFC (*r*=0.52, *P*=0.003). Across all working memory models, there was a significant effect of group in seven endogenous connectivity parameters (all *P*<0.03), which was driven by the difference between patients and the other two groups. Patients showed reduced strength in the reciprocal connections between the right and left DLPFC, in the forward connections from the left and right PAR to the left ACC and right DLPFC, respectively, as well as the connection from the right to the left PAR. Finally, the backward connectivity from the PFC was reduced between left DLPFC and left ACC and between the right DLPFC and right PAR (Figure 3b).

No significant effect of the medication type or dose was found on any connectivity parameters (*P*>0.42) in either paradigm.

## Discussion

In the present study we compared endogenous and modulated connectivity parameters in patients with BD, resilient relatives and unrelated healthy controls during facial affect processing and working memory to identify connectomic markers of genetic risk, resilience and disease expression.

### Connectomic markers of shared genetic risk for BD

In line with previous neuroimaging studies of facial affect processing, we found significantly increased connectivity between the AMG and the VPFC in patients with BD and their unaffected relatives.^19, 25, 26, 27, 38, 59, 60^ This finding therefore represents a connectomic marker of shared genetic vulnerability to the disorder. However, it is not sufficient for disease expression as it was present in relatives who had remained free of any clinical psychopathology. The presence of this shared genetic connectomic abnormality confirms that resilience in the relatives must arise from adaptive neural responses that can overcome their expressed genetic risk.

### Adaptive hyperconnectivity as a marker of resilience to BD

At any given point in time, the connectomic features of a network are defined by the nature and degree of neural network disruption, the demands placed on the network by internal or external context and by the availability of network reserve.^22^ Across all brain disorders, the presence of neural dysfunction results in reduced or lost network connectivity when network reserves are depleted. However, when network resources are still available, increased connectivity is considered the most common response.^22^ Within this framework, the presence of frontolimbic hyperactivation in patients and relatives confirms a shared genetic response to facial affect-processing network dysfunction. However, the additional hyperconnectivity observed only in relatives can be viewed as an adaptive network response-associated greater network reserve. The adaptive nature of this response can be inferred by its association with preserved mental well being in the relatives. Additional support is provided by longitudinal studies in patients with BD where successful treatment is associated with increased connectivity throughout the facial affect-processing network.^61^ Of further significance is the increased endogenous connectivity within the ventral visual stream in resilient relatives who showed increased reciprocal coupling between the IOG and FG. The FG is involved in early perceptual visual processing where it contributes to the categorization of facial identity and valence.^62, 63^ In patients with BD there is reduced FG engagement from very early disease expression^60, 64^ and exaggerated volume loss during disease progression.^65^ It would therefore appear that resilient relatives have adapted their neural responses to emotional faces via additional recruitment throughout the affect-processing network, which is suggestive of increased plasticity between lower and higher visual areas that may increase functional network efficiency.

### Hypoconnectivity as a connectomic marker of disease expression in BD

Our results show that healthy individuals and resilient relatives engaged the same optimal DCM for working memory and did not differ in any connectomic parameter in terms of endogenous connections or modulations. In contrast, no single DCM appeared to explain the working memory network architecture in patients. Moreover, patients showed widespread hypoconnectivity within the entire working memory network. This is consistent with convergent reports of prior neuropsychological and functional neuroimaging studies of working memory dysfunction in patients.^7, 12, 20^ Further, in our study hypoconnectivity within the working memory network was linked to disease severity and functional impairment. Hypoconnectivity between visual and prefrontal regions declined further with increasing illness duration and was associated with lower everyday functioning level. Working memory dysfunction is considered a major contributor to patients' inability to regain a premorbid level of functioning and to ongoing psychosocial impairment.^66, 67^ Although patients were medicated, it is unlikely that medication contributed to this widespread hypoconnectivity as successful treatment with medication has been shown to promote normalization of connectivity deficits across disorders.^68, 69^

Our results with regards to working memory are not dissimilar to findings within the field of schizophrenia where hypoconnectivity is generally observed within the executive control network.^70^ Such an overlap between schizophrenia and BD is often considered in terms of their overlap in polygenic risk scores.^71^ However, recent findings suggest that the working memory network may be particularly sensitive to a diagnosis-independent psychopathology, for example, hypoconnectivity in dorsal prefrontal and parietal regions seems to index higher levels of neuroticism,^72^ a known transdiagnostic risk factor for psychiatric disorders.^73^

### Methodological considerations

A particular strength of the study was the inclusion of a carefully selected group of resilient relatives. Resilience, or health for that matter, cannot be considered immutable traits. It is theoretically possible that the relatives selected may present with psychiatric pathology in some future time. However, this likelihood is generally statistically small and we took steps to ensure that none of the relatives showed any signs of imminent conversion. A conservative view of our results is that the adaptive connectomic signature identified in resilient relatives is, at the very least, associated with very delayed disease onset. We did not examine the polygenic score of relatives because it represents a summary measure of genetic risk to BD that is mechanistically not more informative than family history as it does not allow us to make direct inferences about its association with specific phenotypic and connectomic traits. However, this is an interesting avenue for further research.

## Conclusions

Our findings suggest that resilience to genetic risk of BD may reflect the capacity to adapt network connectivity to ameliorate the effects of underlying network dysfunction. Further neuroimaging studies on adaptive connectivity features to avert the manifestation of BD have the potential to assist in formulating biologically informed preventative strategies and aid in the development of future studies on high-risk populations.^74, 75^

## Figures and Tables

**Table 1:** Demographic, clinical and behavioral data

	*Patients with bipolar disorder (*n=*41)*	*Unrelated healthy controls (*n=*46)*	*Resilient relatives (*n=*25)*
*Demographic variables*			
Age	44.3 (11.9)	40.3 (13.2)	39.7 (13.7)
Sex (male/female)	20/21	25/21	13/12
IQ	117.9 (17.9)	112.6 (14.5)	115.8 (18.5)

*Clinical variables*			
HDRS total score^a^	4.8 (5.3)	0.1 (0.5)	0.14 (0.4)
YMRS total score^a^	1.4 (3.0)	0.2 (0.6)	0.0 (0.0)
BPRS total score^a^	27.5 (4.0)	24.3 (0.7)	24.1 (0.4)
Age of onset (years)	24.7 (8.0)	—	—
Duration of illness (years)	20.2 (10.5)	—	—
Depressive episodes (*n*)	5.7 (7.5)	—	—
Manic episodes (*n*)	5.6 (7.7)	—	—
GAF	75 (14.9)		

*Facial affect-recognition task performance*			
Accuracy (%)	90.3 (4.1)	93.1 (4.8)	90.1 (5.2)
Response time (s)^b^	1.4 (0.20)	1.10 (0.24)	1.09 (0.14)

*Working memory task performance*			
3-Back accuracy (%)^c^	68.9 (26.7)	72.1 (25.1)	90.1 (15.4)
3-Back response time (s)	0.86 (0.34)	0.87 (0.45)	0.73 (0.22)

Abbreviations: BPRS, Brief Psychiatric Rating Scale; GAF, Global Assessment of Functioning; HDRS, Hamilton Depression Rating Scale; *n*, number; s, seconds; YMRS, Young Mania Rating Scale.

Unless otherwise indicated, data are expressed as mean (s.d.).

a Scores for patients are significantly greater than those of both other groups (*P*<0.019).

b Patients had longer mean response times compared with both other groups (*P*<0.009).

c Relatives showed higher accuracy scores compared with both other groups (*P*<0.003).

## References

[bib1] World Health OrganizationThe Global Burden Of Disease: 2004 Update. World Health Organization: Geneva, Switzerland, 2008.

[bib2] Lichtenstein P, Yip BH, Björk C, Pawitan Y, Cannon TD, Sullivan PF et al. Common genetic determinants of schizophrenia and bipolar disorder in Swedish families: a population-based study. Lancet 2009; 373: 234–239. 19150704 10.1016/S0140-6736(09)60072-6 PMC3879718

[bib3] International Schizophrenia Consortium, Purcell SM, Wray NR, Stone JL, Visscher PM, O'Donovan MC, Sullivan PF et al. Common polygenic variation contributes to risk of schizophrenia and bipolar disorder. Nature 2009; 460: 748–752. 19571811 10.1038/nature08185 PMC3912837

[bib4] Frangou S. Brain structural and functional correlates of resilience to bipolar disorder. Front Hum Neurosci 2012; 5: 1–10. 10.3389/fnhum.2011.00184 PMC3277296 22363273

[bib5] Fusar-Poli P, Howes O, Bechdolf A, Borgwardt S. Mapping vulnerability to bipolar disorder: a systematic review and meta-analysis of neuroimaging studies. J Psychiatry Neurosci 2012; 37: 170–184. 22297067 10.1503/jpn.110061 PMC3341409

[bib6] Kempton MJ, Haldane M, Jogia J, Grasby PM, Collier D, Frangou S. Dissociable brain structural changes associated with predisposition, resilience, and disease expression in bipolar disorder. J Neurosci 2009; 29: 10863–10868. 19726644 10.1523/JNEUROSCI.2204-09.2009 PMC6665540

[bib7] Fears SC, Service SK, Kremeyer B, Araya C, Araya X, Bejarano J et al. Multisystem component phenotypes of bipolar disorder for genetic investigations of extended pedigrees. JAMA Psychiatry 2014; 71: 375–387. 24522887 10.1001/jamapsychiatry.2013.4100 PMC4045237

[bib8] Kempton MJ, Salvador Z, Munafò MR, Geddes JR, Simmons A, Frangou S et al. Structural neuroimaging studies in major depressive disorder. Meta-analysis and comparison with bipolar disorder. Arch Gen Psychiatry 2011; 68: 675–690. 21727252 10.1001/archgenpsychiatry.2011.60

[bib9] Fox MD, Snyder AZ, Vincent JL, Corbetta M, Van Essen DC, Raichle ME. The human brain is intrinsically organized into dynamic, anticorrelated functional networks. Proc Natl Acad Sci USA 2005; 102: 9673–9678. 15976020 10.1073/pnas.0504136102 PMC1157105

[bib10] Smith SM, Fox PT, Miller KL, Glahn DC, Fox PM, Mackay CE et al. Correspondence of the brain's functional architecture during activation and rest. Proc Natl Acad Sci USA 2009; 106: 13040–13045. 19620724 10.1073/pnas.0905267106 PMC2722273

[bib11] Arts B, Jabben N, Krabbendam L, van Os J. Meta-analyses of cognitive functioning in euthymic bipolar patients and their first-degree relatives. Psychol Med 2008; 38: 771–785. 17922938 10.1017/S0033291707001675

[bib12] Glahn DC, Almasy L, Barguil M, Hare E, Peralta JM, Kent Jr et al. Neurocognitive endophenotypes for bipolar disorder identified in multiplex multigenerational families. Arch Gen Psychiatry 2010; 67: 168–177. 20124116 10.1001/archgenpsychiatry.2009.184 PMC3401550

[bib13] Lindquist KA, Wager TD, Kober H, Bliss-Moreau E, Barrett LF. The brain basis of emotion: a meta-analytic review. Behav Brain Sci 2012; 35: 121–143. 22617651 10.1017/S0140525X11000446 PMC4329228

[bib14] Niendam TA, Laird AR, Ray KL, Dean YM, Glahn DC, Carter CS. Meta-analytic evidence for a superordinate cognitive control network subserving diverse executive functions. Cogn Affect Behav Neurosci 2012; 12: 241–268. 22282036 10.3758/s13415-011-0083-5 PMC3660731

[bib15] Chen CH, Suckling J, Lennox BR, Ooi C, Bullmore ET. A quantitative meta-analysis of fMRI studies in bipolar disorder. Bipolar Disord 2011; 13: 1–15. 10.1111/j.1399-5618.2011.00893.x 21320248

[bib16] Thermenos HW, Goldstein JM, Milanovic SM, Whitfield-Gabrieli S, Makris N, Laviolette P et al. An fMRI study of working memory in persons with bipolar disorder or at genetic risk for bipolar disorder. Am J Med Genet B Neuropsychiatr Genet 2010; 153B: 120–131. 19418510 10.1002/ajmg.b.30964 PMC3762486

[bib17] Delvecchio G, Fossati P, Boyer P, Brambilla P, Falkai P, Gruber O et al. Common and distinct neural correlates of emotional processing in Bipolar Disorder and Major Depressive Disorder: a voxel-based meta-analysis of functional magnetic resonance imaging studies. Eur Neuropsychopharmacol 2012; 22: 100–113. 21820878 10.1016/j.euroneuro.2011.07.003

[bib18] Delvecchio G, Sugranyes G, Frangou S. Evidence of diagnostic specificity in the neural correlates of facial affect processing in bipolar disorder and schizophrenia: a meta-analysis of functional imaging studies. Psychol Med 2013; 43: 553–569. 22874625 10.1017/S0033291712001432

[bib19] Dima D, Jogia J, Collier D, Vassos E, Burdick KE, Frangou S. Independent modulation of engagement and connectivity of the facial network during affect processing by CACNA1C and ANK3 risk genes for bipolar disorder. JAMA Psychiatry 2013; 70: 1303–1311. 24108394 10.1001/jamapsychiatry.2013.2099

[bib20] Jogia J, Dima D, Kumari V, Frangou S. Frontopolar cortical inefficiency may underpin reward and working memory dysfunction in bipolar disorder. World J Biol Psychiatry 2012; 13: 605–615. 21812622 10.3109/15622975.2011.585662

[bib21] Drapier D, Surguladze S, Marshall N, Schulze K, Fern A, Hall MH et al. Genetic liability for bipolar disorder is characterized by excess frontal activation in response to a working memory task. Biol Psychiatry 2008; 64: 513–520. 18571627 10.1016/j.biopsych.2008.04.038

[bib22] Hillary FG, Roman CA, Venkatesan U, Rajtmajer SM, Bajo R, Castellanos ND. Hyperconnectivity is a fundamental response to neurological disruption. Neuropsychology 2015; 29: 59–75. 24933491 10.1037/neu0000110

[bib23] Lui S, Yao L, Xiao Y, Keedy SK, Reilly JL, Keefe RS et al. Resting-state brain function in schizophrenia and psychotic bipolar probands and their first-degree relatives. Psychol Med 2015; 45: 97–108. 25066779 10.1017/S003329171400110X PMC5836742

[bib24] Yip SW, Mackay CE, Goodwin GM. Increased temporo-insular engagement in unmedicated bipolar II disorder: an exploratory resting state study using independent component analysis. Bipolar Disord 2014; 16: 748–755. 24725219 10.1111/bdi.12206

[bib25] Almeida JR, Versace A, Mechelli A, Hassel S, Quevedo K, Kupfer DJ et al. Abnormal amygdala-prefrontal effective connectivity to happy faces differentiates bipolar from major depression. Biol Psychiatry 2009; 66: 451–459. 19450794 10.1016/j.biopsych.2009.03.024 PMC2740996

[bib26] Horacek J, Mikolas P, Tintera J, Novak T, Palenicek T, Brunovsky M et al. Sad mood induction has an opposite effect on amygdala response to emotional stimuli in euthymic patients with bipolar disorder and healthy controls. J Psychiatry Neurosci 2015; 40: 134–142. 25703646 10.1503/jpn.140044 PMC4354819

[bib27] Versace A, Thompson WK, Zhou D, Almeida JR, Hassel S, Klein CR et al. Abnormal left and right amygdala-orbitofrontal cortical functional connectivity to emotional faces: state versus trait vulnerability markers of depression in bipolar disorder. Biol Psychiatry 2010; 67: 422–431. 20159144 10.1016/j.biopsych.2009.11.025 PMC2835157

[bib28] Chepenik LG, Raffo M, Hampson M, Lacadie C, Wang F, Jones MM et al. Functional connectivity between ventral prefrontal cortex and amygdala at low frequency in the resting state in bipolar disorder. Psychiatry Res 2010; 182: 207–210. 20493671 10.1016/j.pscychresns.2010.04.002 PMC2914819

[bib29] Radaelli D, Sferrazza Papa G, Vai B, Poletti S, Smeraldi E, Colombo C et al. Fronto-limbic disconnection in bipolar disorder. Eur Psychiatry 2015; 30: 82–88. 24853295 10.1016/j.eurpsy.2014.04.001

[bib30] Roberts G, Green MJ, Breakspear M, McCormack C, Frankland A, Wright A et al. Reduced inferior frontal gyrus activation during response inhibition to emotional stimuli in youth at high risk of bipolar disorder. Biol Psychiatry 2013; 74: 55–61. 23245750 10.1016/j.biopsych.2012.11.004

[bib31] Stegmayer K, Usher J, Trost S, Henseler I, Tost H, Rietschel M et al. Disturbed cortico-amygdalar functional connectivity as pathophysiological correlate of working memory deficits in bipolar affective disorder. Eur Arch Psychiatry Clin Neurosci 2015; 265: 303–301. 25119145 10.1007/s00406-014-0517-5

[bib32] Baker JT, Holmes AJ, Masters GA, Yeo BT, Krienen F, Buckner RL et al. Disruption of cortical association networks in schizophrenia and psychotic bipolar disorder. JAMA Psychiatry 2014; 71: 109–118. 24306091 10.1001/jamapsychiatry.2013.3469 PMC4435541

[bib33] Oertel-Knöchel V, Reinke B, Matura S, Prvulovic D, Linden DE, van de Ven V. Functional connectivity pattern during rest within the episodic memory network in association with episodic memory performance in bipolar disorder. Psychiatry Res 2015; 231: 141–150. 25575881 10.1016/j.pscychresns.2014.11.014

[bib34] Pompei F, Jogia J, Tatarelli R, Girardi P, Rubia K, Kumari V et al. Familial and disease specific abnormalities in the neural correlates of the Stroop Task in Bipolar Disorder. Neuroimage 2011; 56: 1677–1684. 21352930 10.1016/j.neuroimage.2011.02.052

[bib35] Pompei F, Dima D, Rubia K, Kumari V, Frangou S. Dissociable functional connectivity changes during the Stroop task relating to risk, resilience and disease expression in bipolar disorder. Neuroimage 2011; 57: 576–582. 21570470 10.1016/j.neuroimage.2011.04.055

[bib36] Rashid B, Damaraju E, Pearlson GD, Calhoun VD. Dynamic connectivity states estimated from resting fMRI Identify differences among Schizophrenia, bipolar disorder, and healthy control subjects. Front Hum Neurosci 2014; 8: 897. 25426048 10.3389/fnhum.2014.00897 PMC4224100

[bib37] Samudra N, Ivleva EI, Hubbard NA, Rypma B, Sweeney JA, Clementz BA et al. Alterations in hippocampal connectivity across the psychosis dimension. Psychiatry Res 2015; 233: 148–157. 26123450 10.1016/j.pscychresns.2015.06.004 PMC4784701

[bib38] Manelis A, Ladouceur CD, Graur S, Monk K, Bonar LK, Hickey MB et al. Altered amygdala-prefrontal response to facial emotion in offspring of parents with bipolar disorder. Brain 2015; 138: 2777–2790. 26112339 10.1093/brain/awv176 PMC4643621

[bib39] Diwadkar VA, Bakshi N, Gupta G, Pruitt P, White R, Eickhoff SB. Dysfunction and dysconnection in cortical-striatal networks during sustained attention: genetic risk for schizophrenia or bipolar disorder and its impact on brain network function. Front Psychiatry 2014; 5: 50. 24847286 10.3389/fpsyt.2014.00050 PMC4023040

[bib40] Friston KJ, Harrison L, Penny W. Dynamic causal modelling. Neuroimage 2003; 19: 1273–1302. 12948688 10.1016/s1053-8119(03)00202-7

[bib41] Frangou S. Risk and resilience in bipolar disorder: rationale and design of the Vulnerability to Bipolar Disorders Study (VIBES). Biochem Soc Trans 2009; 37: 1085–1089. 19754457 10.1042/BST0371085

[bib42] First MB, Spitzer RL, Gibbon M, Williams JBW. Structured Clinical Interview for DSM-IV-TR Axis I Disorders, Research Version, Patient Edition, (SCID-I/P). New York Biometrics Research: New York, NY, USA, 2002a.

[bib43] First MB, Spitzer RL, Gibbon M, Williams JBW. Structured Clinical Interview for DSM-IV-TR Axis I Disorders, Research Version, Non-Patient Edition, (SCID-I/NP). New York Biometrics Research: New York, NY, USA, 2002b.

[bib44] American Psychiatric AssociationDiagnostic and Statistical Manual of Mental Disorders. 4th edn, American Psychiatric Association: Washington, DC, USA, 1994.

[bib45] Hamilton M. A rating scale for depression. J Neurol Neurosurg Psychiatry 1960; 23: 56–62. 14399272 10.1136/jnnp.23.1.56 PMC495331

[bib46] Young RC, Biggs JT, Ziegler VE, Meyer DA. A rating scale for mania: reliability, validity and sensitivity. Br J Psychiatry 1978; 133: 429–435. 728692 10.1192/bjp.133.5.429

[bib47] Lukoff D, Liberman RP, Nuechterlien KH. Symptom monitoring in the rehabilitation of schizophrenic patients. Schizophr Bull 1986; 12: 578–602. 3810065 10.1093/schbul/12.4.578

[bib48] Wechsler D. Wechsler Adult Intelligence Scale - Third Edition. The Psychological Corporation: San Antonio, USA, 1997.

[bib49] Merikangas KR, Jin R, He JP, Kessler RC, Lee S, Sampson NA et al. Prevalence and correlates of bipolar spectrum disorder in the world mental health survey initiative. Arch Gen Psychiatry 2011; 68: 241–251. 21383262 10.1001/archgenpsychiatry.2011.12 PMC3486639

[bib50] Coryell W, Fiedorowicz J, Leon AC, Endicott J, Keller MB. Age of onset and the prospectively observed course of illness in bipolar disorder. J Affect Disord 2013; 146: 34–38. 23062746 10.1016/j.jad.2012.08.031 PMC3605729

[bib51] Talairach J, Tournoux P. Co-planar Stereotaxic Atlas of the Human Brain: 3-Dimensional Proportional System—an Approach to Cerebral Imaging. Thieme Medical Publishers: New York, NY, USA, 1988.

[bib52] Dima D, Stephan KE, Roiser JP, Friston KJ, Frangou S. Effective connectivity during processing of facial affect: evidence for multiple parallel pathways. J Neurosci 2011; 31: 14378–14385. 21976523 10.1523/JNEUROSCI.2400-11.2011 PMC6623650

[bib53] Fairhall SL, Ishai A. Effective connectivity within the distributed cortical network for face perception. Cereb Cortex 2007; 17: 2400–2406. 17190969 10.1093/cercor/bhl148

[bib54] Torrisi SJ, Lieberman MD, Bookheimer SY, Altshuler LL. Advancing understanding of affect labeling with dynamic causal modeling. Neuroimage 2013; 82: 481–488. 23774393 10.1016/j.neuroimage.2013.06.025 PMC3759566

[bib55] Owen AM, McMillan KM, Laird AR, Bullmore ET. N-back working memory paradigm: a meta-analysis of normative functional neuroimaging studies. Hum Brain Mapp 2005; 25: 46–59. 15846822 10.1002/hbm.20131 PMC6871745

[bib56] Dima D, Jogia J, Frangou S. Dynamic causal modeling of load-dependent modulation of effective connectivity within the verbal working memory network. Hum Brain Mapp 2014; 35: 3025–3035. 24142432 10.1002/hbm.22382 PMC6869395

[bib57] Stephan KE, Penny WD, Moran RJ, den Ouden HEM, Daunizeau J, Friston KJ. Ten simple rules for dynamic causal modeling. Neuroimage 2010; 49: 3099–3109. 19914382 10.1016/j.neuroimage.2009.11.015 PMC2825373

[bib58] Penny WD, Stephan KE, Daunizeau J, Rosa MJ, Friston KJ, Schofield TM et al. Comparing families of dynamic causal models. PLoS Comput Biol 2010; 6: e1000709. 20300649 10.1371/journal.pcbi.1000709 PMC2837394

[bib59] Kanske P, Schönfelder S, Forneck J, Wessa M. Impaired regulation of emotion: neural correlates of reappraisal and distraction in bipolar disorder and unaffected relatives. Transl Psychiatry 2015; 5: e497. 25603413 10.1038/tp.2014.137 PMC4312831

[bib60] Perlman SB, Fournier JC, Bebko G, Bertocci MA, Hinze AK, Bonar L et al. Emotional face processing in pediatric bipolar disorder: evidence for functional impairments in the fusiform gyrus. J Am Acad Child Adolesc Psychiatry 2013; 52: 1314–1325. 24290464 10.1016/j.jaac.2013.09.004 PMC3881180

[bib61] Vai B, Poletti S, Radaelli D, Dallaspezia S, Bulgarelli C, Locatelli C et al. Successful antidepressant chronotherapeutics enhance fronto-limbic neural responses and connectivity in bipolar depression. Psychiatry Res 2015; 233: 243–253. 26195295 10.1016/j.pscychresns.2015.07.015

[bib62] Said CP, Haxby JV, Todorov A. Brain systems for assessing the affective value of faces. Philos Trans R Soc Lond B Biol Sci 2011; 366: 1660–1670. 21536552 10.1098/rstb.2010.0351 PMC3130375

[bib63] Vuilleumier P, Pourtois G. Distributed and interactive brain mechanisms during emotion face perception: evidence from functional neuroimaging. Neuropsychologia 2007; 45: 174–194. 16854439 10.1016/j.neuropsychologia.2006.06.003

[bib64] Adleman NE, Kayser RR, Olsavsky AK, Bones BL, Muhrer EJ, Fromm SJ et al. Abnormal fusiform activation during emotional-face encoding assessed with functional magnetic resonance imaging. Psychiatry Res 2013; 212: 161–163. 23541333 10.1016/j.pscychresns.2013.01.006 PMC3717571

[bib65] Moorhead TW, McKirdy J, Sussmann JE, Hall J, Lawrie SM, Johnstone EC et al. Progressive gray matter loss in patients with bipolar disorder. Biol Psychiatry 2007; 62: 894–900. 17617385 10.1016/j.biopsych.2007.03.005

[bib66] Bearden CE, Woogen M, Glahn DC. Neurocognitive and neuroimaging predictors of clinical outcome in bipolar disorder. Curr Psychiatry Rep 2010; 12: 499–504. 20839077 10.1007/s11920-010-0151-5 PMC2965363

[bib67] Solé B, Bonnin CM, Torrent C, Martinez-Aran A, Popovic D, Tabarés-Seisdedos R et al. Neurocognitive impairment across the bipolar spectrum. CNS Neurosci Ther 2012; 18: 194–200. 22128808 10.1111/j.1755-5949.2011.00262.x PMC6493522

[bib68] Sarpal DK, Robinson DG, Lencz T, Argyelan M, Ikuta T, Karlsgodt K et al. Antipsychotic treatment and functional connectivity of the striatum in first-episode schizophrenia. JAMA Psychiatry 2015; 72: 5–13. 25372846 10.1001/jamapsychiatry.2014.1734 PMC4286512

[bib69] Schmidt A, Smieskova R, Aston J, Simon A, Allen P, Fusar-Poli P et al. Brain connectivity abnormalities predating the onset of psychosis: correlation with the effect of medication. JAMA Psychiatry 2013; 70: 903–912. 23824230 10.1001/jamapsychiatry.2013.117

[bib70] Fornito A, Zalesky A, Pantelis C, Bullmore ET. Schizophrenia, neuroimaging and connectomics. Neuroimage 2012; 62: 2296–2314. 22387165 10.1016/j.neuroimage.2011.12.090

[bib71] Maier R, Moser G, Chen GB, Ripke S, Cross-Disorder Working Group of the Psychiatric Genomics Consortium, Coryell W et al. Joint analysis of psychiatric disorders increases accuracy of risk prediction for schizophrenia, bipolar disorder, and major depressive disorder. Am J Hum Genet 2015; 96: 283–294. 25640677 10.1016/j.ajhg.2014.12.006 PMC4320268

[bib72] Dima D, Friston KJ, Stephan KE, Frangou S. Neuroticism and conscientiousness respectively constrain and facilitate short-term plasticity within the working memory neural network. Hum Brain Mapp 2015; 36: 4158–4163. 26189566 10.1002/hbm.22906 PMC4863074

[bib73] Caspi A, Houts RM, Belsky DW, Goldman-Mellor SJ, Harrington H, Israel S et al. The p factor: one general psychopathology factor in the structure of psychiatric disorders? Clin Psychol Sci 2014; 2: 119–137. 25360393 10.1177/2167702613497473 PMC4209412

[bib74] Brotman MA, Deveney CM, Thomas LA, Hinton KE, Yi JY, Pine DS et al. Parametric modulation of neural activity during face emotion processing in unaffected youth at familial risk for bipolar disorder. Bipolar Disord 2014; 16: 756–763. 24617738 10.1111/bdi.12193 PMC4162856

[bib75] Singh MK, Chang KD, Kelley RG, Saggar M, Reiss AL, Gotlib IH. Early signs of anomalous neural functional connectivity in healthy offspring of parents with bipolar disorder. Bipolar Disord 2014; 16: 678–689. 24938878 10.1111/bdi.12221 PMC4213354

