# Supplementary material for: Connectomic markers of disease expression, genetic risk and resilience in bipolar disorder
Source: Transl Psychiatry. 2016 Jan 5;6(1):e706–. doi: 10.1038/tp.2015.193 (PMC5068872; doi:10.1038/tp.2015.193)
Supplement: Supplementary Information [file tp2015193x1.doc]

**Supplemental Information**

**Methods**

**Dynamic Causal Modelling**

DCM tests a set of models and, through Bayesian model selection, provides evidence in favor of one model, relative to others. In DCM regional blood-oxygen-dependent responses are modeled by a bilinear differential equation that describes how the underlying neural states change as a function of endogenous connections between regions (referred to as nodes), modulatory effects on these connections, and driving inputs (Friston et al., 2003). Endogenous connections refer to the coupling strength between nodes in the absence of any input to the network (task-independent). Modulatory effects refer to task-dependent changes in coupling strength. The driving input models how task-related information enters the network. For each task we defined the relevant model space (i.e., the set of models that are plausible) based on current best evidence regarding the neural circuitry that supports facial affect recognition and working memory.

For the *facial affect recognition* paradigm, the spatial part of the basic 4- node model was specified as four VOIs, each of 5mm radius, centered on the coordinates of the group maxima of the contrast affective > neutral faces from the entire study sample: IOG (x=44, y=-76 , z=-1), FG (x=24, y=-55, z=-7), AMG (x=16, y=-3, z=-13) and VPFC (x=51, y=25, z=-6).

For the *working memory* paradigm, the spatial part of the basic 8-node model was specified as 8 VOIs, each of 5mm radius, centered on the coordinates of the group maxima of the contrast 1-, 2-, 3 -back > 0-back condition (following conjunction analysis) from the entire study sample: IOG (x=-40, y=-68, z=-5 and x=44, y=-78, z=-6), PAR (x=-36, y=-52, z=45 and x=40, y=-44, z=43), ACC (x=10, y=21, z=25 and x=10, y=28, z=24), and DLPFC (x=-46, y=35, z=30 and x=44, y=38, z=31).

For each paradigm, the spatial models were further refined for each participant based on the participant-specific maxima that were: (i) within 4 mm from the group maxima, (ii) within the same anatomical regions, as defined by the PickAtlas toolbox (http://www.nitrc.org/projects/wfu_pickatlas/) and (iii) adjusted using the effect of interest F-contrast. Regional time series were summarized with the first eigenvariate of all activated (at p<0.01) voxels within participant-specific VOIs.

Friston KJ, Harrison L, Penny W. Dynamic causal modelling. *Neuroimage* 2003; **19**: 1273-1302.

**Supplemental Figure S1.** **Seven dynamic causal models for the face affect paradigm for bipolar disorder patients (BD), their resilient relatives and healthy individuals.** The model is compromised of four brain areas specified with bidirectional endogenous connections between all regions (inferior occipital gyrus = IOG, fusiform gyrus = FG, amygdala = AMG, ventral prefrontal cortex = VPFC; all located in the right hemisphere) and with a driving input of ‘all faces’ into the IOG. Green lines represents the affect faces modulation.

| Supplemental Table S1. Dynamic Causal Modelling: Specification of Model Architecture | |
| --- | --- |
| **Model** | **Modulation on:** |
| ***Face affect paradigm - Facial affect modulation*** | |
| **Model 1** | IOG → VPFC |
| **Model 2** | FG → VPFC |
| **Model 3** | AMG → VPFC |
| **Model 4** | IOG → VPFC; FG → VPFC; AMG → VPFC |
| **Model 5** | IOG → VPFC; FG → VPFC |
| **Model 6** | FG → VPFC; AMG → VPFC |
| **Model 7** | IOG → VPFC; AMG → VPFC |
| ***Working memory paradigm - 3-back modulation*** | |
| **Model 1** | lIOG → rIOG |
| **Model 2** | rIOG → lIOG |
| **Model 3** | lIOG → lPAR |
| **Model 4** | lPAR → lIOG |
| **Model 5** | lIOG → lACC |
| **Model 6** | lACC → lIOG |
| **Model 7** | lIOG → lDLPFC |
| **Model 8** | lDLPFC → lIOG |
| **Model 9** | lPAR → rPAR |
| **Model 10** | rPAR → lPAR |
| **Model 11** | lPAR → lACC |
| **Model 12** | lACC → lPAR |
| **Model 13** | lPAR → lDLPFC |
| **Model 14** | lDLPFC → lPAR |
| **Model 15** | lACC → lDLPFC |
| **Model 16** | lDLPFC → lACC |
| **Model 17** | lDLPFC → rDLPFC |
| **Model 18** | rDLPFC → lDLPFC |
| **Model 19** | lACC → rACC |
| **Model 20** | rACC → lACC |
| **Model 21** | rACC → rDLPFC |
| **Model 22** | rDLPFC → rACC |
| **Model 23** | rDLPFC→ rPAR |
| **Model 24** | rPAR → rDLPFC |
| **Model 25** | rDLPFC → rIOG |
| **Model 26** | rIOG → rDLPFC |
| **Model 27** | rIOG → rACC |
| **Model 28** | rACC → rIOG |
| **Model 29** | rPAR → rACC |
| **Model 30** | rACC → rPAR |
| **Model 31** | rPAR → rIOG |
| **Model 32** | rIOG → rPAR |
| **ACC=Anterior Cingulate Cortex; AMG=Amygdala; DLPFC=Dorsolateral Prefrontal Cortex; FG=Fusiform Gyrus; IOG=Inferior Occipital Gyrus; PAR=Parietal Cortex; VPFC=Ventral Prefrontal Cortex; l=left; r=right; arrow indicates direction of connectivity modulation** | |

**
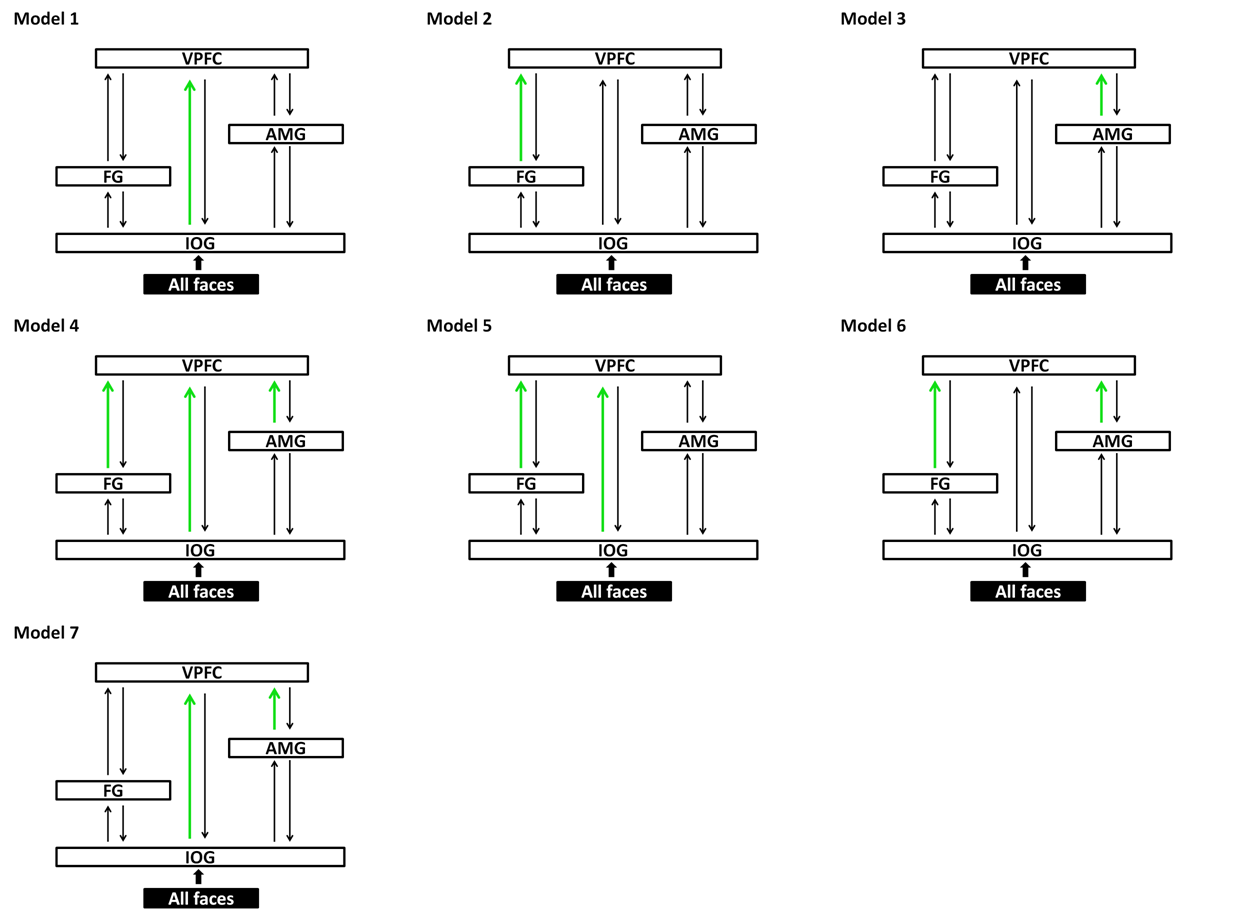
**

**Supplemental Figure 2.** **Thirty-two dynamic causal models for the working memory paradigm for bipolar disorder patients (BD), their resilient relatives and healthy individuals.** An eight-area DCM was specified with bidirectional endogenous connections between all brain regions (lIOG = left IOG and rIOG = right IOG; lPAR = left parietal cortex and rPAR = right PAR; lACC = left anterior cingulate cortex and rACC = right ACC; lDLPFC = left dorsolateral prefrontal cortex and rDLPFC = right DLPFC) in each hemisphere and lateral connections between homologous areas. Driving input of ‘1, 2, 3 -back’ modelled into the left and right IOG. Green lines represents the 3-back modulation.


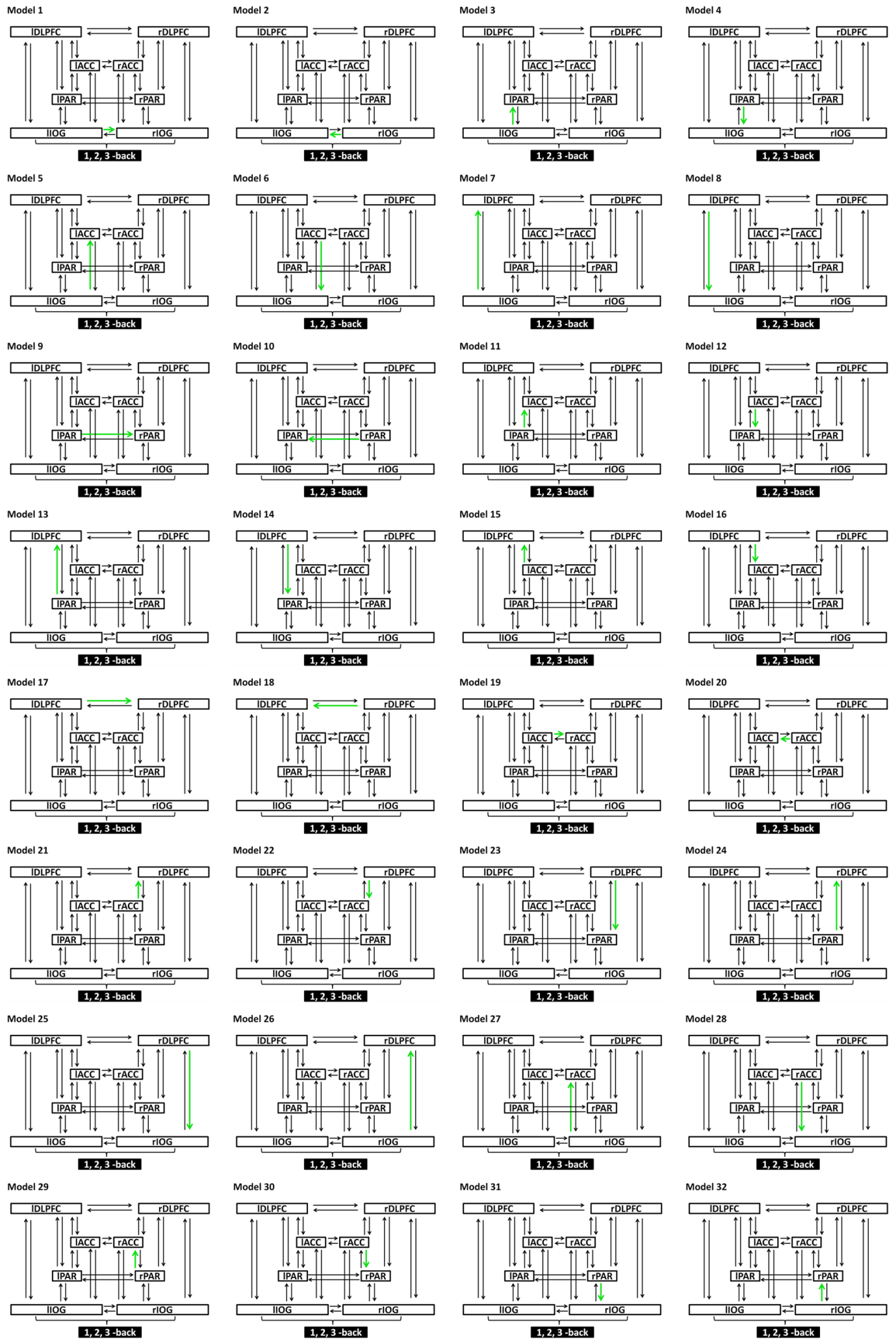


**Supplemental Information**

**Results**

Conventional fMRI

| **Supplemental Table S2. Brain regions showing significant effects of group (patients with bipolar disorder, resilient relatives, unrelated healthy controls) during facial affect processing and working memory (all p < 0.05; family wise error corrected)** | | | | | | | |
| --- | --- | --- | --- | --- | --- | --- | --- |
| **Region** |  | **Laterality** | **Brodmann Area** | **Talairach and Tournoux Coordinates** | | | **z-value** |
|  |  |  |  | **x** | **y** | **z** |  |

|  | | | | | | |
| --- | --- | --- | --- | --- | --- | --- |
| ***Facial Affect Processing (affective faces > neutral faces)*** | | | | | | |
| **Anterior Cingulate Gyrus** | Right | 24 | 4 | 33 | -1 | 3.23 |
| **Superior Frontal**  **Gyrus** | Right | 6 | 38 | 20 | 58 | 3.12 |
| ***Working memory paradigm (3-back > 0-back)*** | | | | | | |

| Middle Frontal  Gyrus  Inferior Frontal  Gyrus  Middle Temporal  Gyrus  Anterior cingulate Gyrus | Right  Left  Left  Right  Right  Left  Right | 9  9  46  46  21  24/32  24/32 | 40  -48  -46  42  67  -14  10 | 18  29  36  11  -7  46  26 | 51  34  26  25  -9  6  -6 | 5.91  4.76  5.41  5.19  4.53  3.56  3.49 |
| --- | --- | --- | --- | --- | --- | --- |

**Dynamic Causal Modelling**

Model selection for each paradigm identified the optimal model for each group as the model with the highest evidence and the best balance between accuracy and complexity and is expressed in terms of exceedance probability, a measure that is very robust to potential outliers. The exceedance probabilities of all models testes are detailed in Supplemental table S3 below.

| **Supplemental Table S3. Exceedance Probabilities per group and paradigm** | | | |
| --- | --- | --- | --- |
|  | **Controls** | **Relatives** | **Patients** |
| Facial Affect Processing | | | |
| **Model 1** | 41 | 14 | 17 |
| **Model 2** | 6 | 16 | 19 |
| **Model 3** | 18 | 8 | 32 |
| **Model 4** | 15 | 33 | 8 |
| **Model 5** | 6 | 16 | 6 |
| **Model 6** | 4 | 8 | 14 |
| **Model 7** | 10 | 5 | 4 |
| **Working Memory** | | | |
| **Model 1** | 1 | 3 | 2 |
| **Model 2** | 1 | 2 | 5 |
| **Model 3** | 2 | 3 | 6 |
| **Model 4** | 1 | 3 | 3 |
| **Model 5** | 1 | 3 | 1 |
| **Model 6** | 1 | 3 | 2 |
| **Model 7** | 3 | 3 | 2 |
| **Model 8** | 1 | 2 | 2 |
| **Model 9** | 3 | 2 | 4 |
| **Model 10** | 1 | 2 | 1 |
| **Model 11** | 1 | 3 | 2 |
| **Model 12** | 1 | 2 | 2 |
| **Model 13** | 1 | 3 | 4 |
| **Model 14** | 1 | 2 | 1 |
| **Model 15** | 1 | 2 | 1 |
| **Model 16** | 1 | 3 | 5 |
| **Model 17** | 1 | 3 | 4 |
| **Model 18** | 2 | 3 | 1 |
| **Model 19** | 1 | 3 | 2 |
| **Model 20** | 1 | 3 | 1 |
| **Model 21** | 3 | 3 | 5 |
| **Model 22** | 1 | 2 | 5 |
| **Model 23** | 1 | 2 | 1 |
| **Model 24** | 1 | 3 | 5 |
| **Model 25** | 3 | 2 | 5 |
| **Model 26** | 57 | 20 | 8 |
| **Model 27** | 1 | 2 | 5 |
| **Model 28** | 1 | 2 | 3 |
| **Model 29** | 1 | 2 | 1 |
| **Model 30** | 1 | 2 | 2 |
| **Model 31** | 1 | 2 | 3 |
| **Model 32** | 3 | 5 | 6 |
